# Non-invasive *in vivo* determination of viable islet graft volume by ^111^In-exendin-3

**DOI:** 10.1038/s41598-017-07815-3

**Published:** 2017-08-03

**Authors:** Wael A. Eter, Inge Van der Kroon, Karolina Andralojc, Mijke Buitinga, Stefanie M. A. Willekens, Cathelijne Frielink, Desiree Bos, Lieke Joosten, Otto C. Boerman, Maarten Brom, Martin Gotthardt

**Affiliations:** Department of Radiology and Nuclear Medicine, Radboud University Medical Centre, Nijmegen, The Netherlands

## Abstract

Pancreatic islet transplantation is a promising therapy for patients with type 1 diabetes. However, the duration of long-term graft survival is limited due to inflammatory as well as non-inflammatory processes and routine clinical tests are not suitable to monitor islet survival. ^111^In-exendin-SPECT (single photon emission computed tomography) is a promising method to non-invasively image islets after transplantation and has the potential to help improve the clinical outcome. Whether ^111^In-exendin-SPECT allows detecting small differences in beta-cell mass (BCM) and measuring the actual volume of islets that were successfully engrafted has yet to be demonstrated. Here, we evaluated the performance of ^111^In-exendin-SPECT using an intramuscular islet transplantation model in C3H mice. *In vivo* imaging of animals transplanted with 50, 100, 200, 400 and 800 islets revealed an excellent linear correlation between SPECT quantification of ^111^In-exendin uptake and insulin-positive area of islet transplants, demonstrating that ^111^In-exendin-SPECT specifically and accurately measures BCM. The high sensitivity of the method allowed measuring small differences in graft volumes, including grafts that contained less than 50 islets. The presented method is reliable, convenient and holds great potential for non-invasive monitoring of BCM after islet transplantation in humans.

## Introduction

Transplantation of islets of Langerhans is a promising treatment for patients with type 1 diabetes (T1D). The short-term results of islet transplantation in normalizing blood glucose levels are encouraging^1^. However, the rate of insulin-independency drops to less than 15% after 5 years^2^. Although the loss of transplanted beta-cells quickly renders patients insulin-dependent in a life-time perspective, the remaining graft function still exerts a positive effect on glucose homeostasis, reducing late complications and further progression of micro-vascular diseases^3^. Furthermore, the islet grafts lower the amount of insulin required to maintain normoglycemia preventing potentially lethal, severe hypoglycemia. However, in view of the considerable side effects caused by the immunosuppressive therapy required to prevent graft rejection, improved survival of the transplanted islet is desirable. In order to optimize islet replacement therapy and to prevent loss of graft function, numerous approaches are currently under investigation, including modified immunosuppressive treatments^4, 5^ and islet encapsulation strategies^6, 7^ treatment with growth factors and other hormones, as well as alternative sources for beta-cells (i.e. stem cells, tissue bioengineering)^8–11^. In order to monitor the graft volume and optimize new strategies for beta-cells replacement a non-invasive technology to visualize viable transplanted islets *in vivo* is warranted. Such a method should be quantitative and sensitive in order to allow the detection of small changes in the number of surviving islets.

A promising approach to visualize transplanted islets *in vivo* has been demonstrated by Saudek *et al*., using Magnetic Resonance Imaging (MRI). The group described MRI of islets that had been labeled with super-paramagnetic iron oxide particles (SPIOs) prior to transplantation^12^. The feasibility of longitudinal non-invasive monitoring of islet transplants was further demonstrated in animal models by Evgenov and co-workers, successfully monitoring islet transplants for up to 188 days after surgery^13^. Pre-labeling of the islets with SPIOs is therefore a promising method with clinical potential^14^.

Alternatively, radionuclide imaging modalities were used because of their high detection sensitivity of transplanted islets. First results of PET imaging of an islet graft were published in 2006, using islets which were transfected with an insulin promoter-dependent reporter gene that leads to trapping of the PET probe ^18^F-FHBG in islet grafts^15^. In another experiment, islets were pre-labeled with ^18^F-fluorodeoxyglucose (^18^F-FDG) and post-transplantation events could be monitored for up to 6 hours after transplantation^16^.

More recently, specific targeting of beta-cells after *in vivo* injection of radiolabeled exendin followed by SPECT imaging was reported as a promising strategy to non-invasively visualize and quantify BCM in the pancreas of rodents, as well as in healthy and diabetic individuals^17, 18^. Similar exendin-based radiotracers were applied for non-invasive imaging of islet grafts in rodent transplantation models as well as in human skeletal muscle^19–21^. Although the use of such tracers in a clinical setting of islet transplantation is highly warranted, establishing the correlation between true BCM and the uptake of the radiotracer *in vivo* is the essential validation step before such studies should be conducted in humans. Such decisive studies have not been performed for GLP-1R imaging of islet transplants.

In the present study, we measured the uptake of ^111^In-exendin-3 in transplants consisting of different amounts of islets in the calf muscles of C3H mice by non-invasive SPECT imaging *in vivo* and validated ^111^In-exendin as a quantitative biomarker for assessment of transplanted beta-cell volume.

## Results

### ^111^In-exendin accumulation in the muscle co-localizes with islet transplants

**Figure 1:**
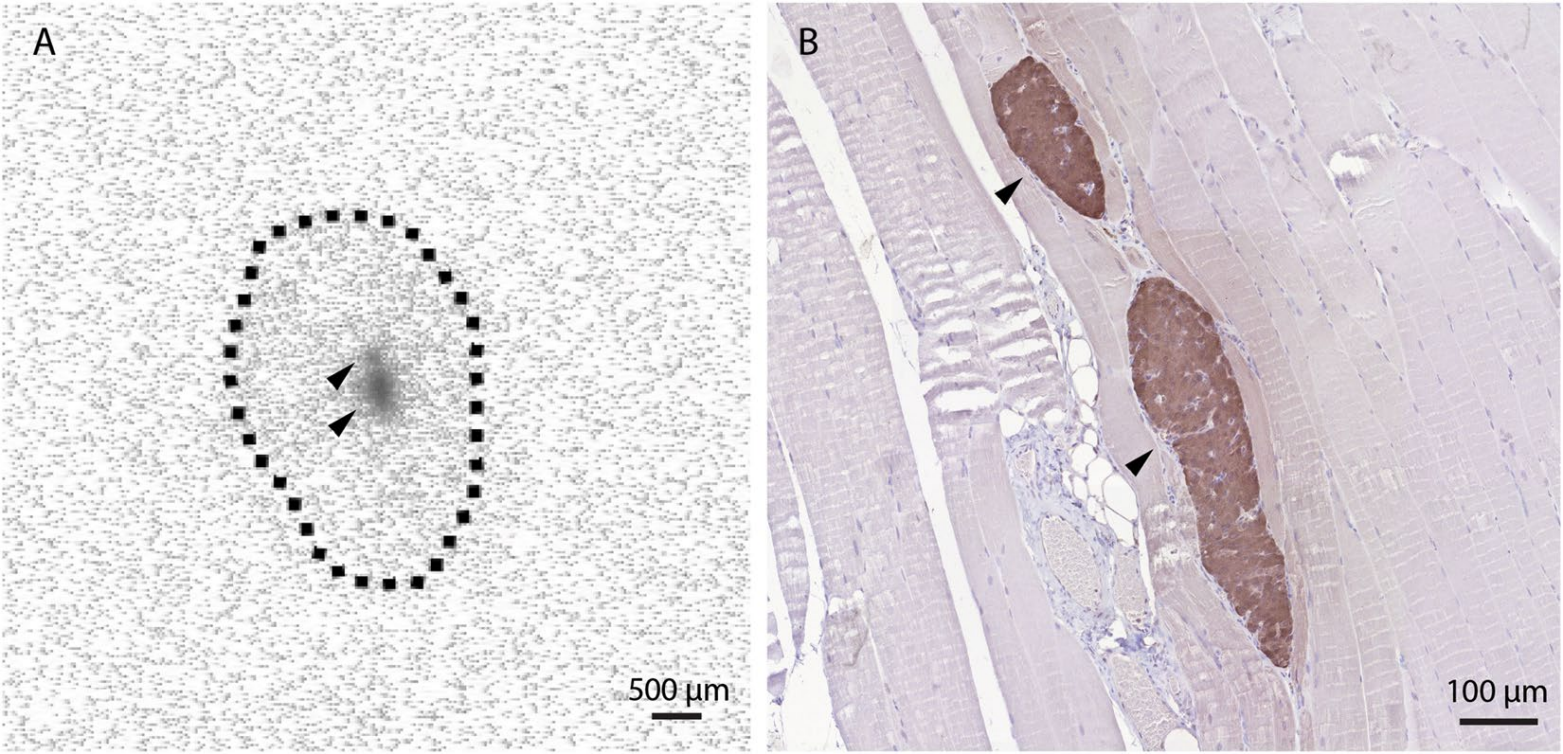
^111^In-exendin-3 uptake in the skeletal muscle is co-localized with islet transplants. (**A**) Autoradiography of muscle sections shows local accumulation of ^111^In-exendin-3 (black arrows), the limits of the muscle section are shown by the dashed line. (**B**) Immunostaining of the corresponding autoradiography section shows co-localization between beta-cells (brown) and ^111^In-exendin-3.

To check whether ^111^In-exendin-3 signal originates from transplanted islets, C3H mice were transplanted with 800 islets in the calf muscle and were injected with ^111^In-exendin-3 four weeks after transplantation, where accumulation of the radiotracer in the islets becomes reproducible^22^. Autoradiographical analysis of muscle sections showed tracer accumulation in well localized regions of the tissue (Fig. 1A) and immunostaining for insulin confirmed that the radioactive signal originated from the islets (Fig. 1B).

### ^111^In-exendin uptake by transplanted islets correlates linearly with BCM

**Figure 2:**
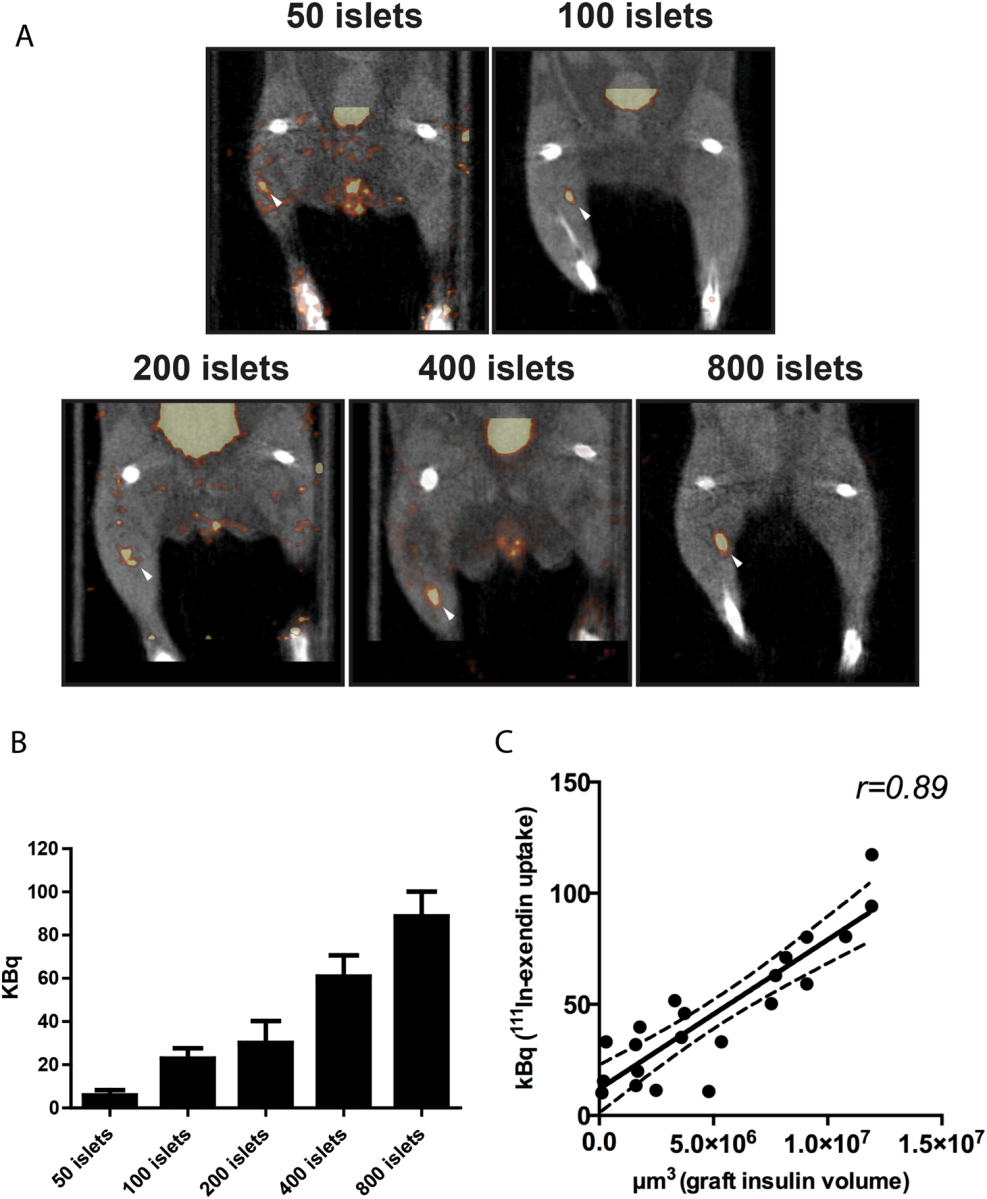
Uptake of ^111^In-exendin by islet transplants is in linear correlation with transplant size. (**A**) SPECT imaging detected the grafts initially transplanted with 50, 100, 200, 400 or 800 islets with high sensitivity, 4 weeks after transplantation (white arrows). (**B**) ^111^In-exendin uptake (expressed in kilobecquerels, kBq) was dependent on transplant size (n = 4–5). Data are shown as means ± SEM (n = 5). (**C**) SPECT signal (expressed in kBq) correlated linearly with graft insulin-positive volume (µm^3^). (Pearson *r* = 0.89, 95% confidence interval shown as a dotted line).

^111^In-exendin-3 uptake in grafts containing various amounts of islets was detected and clearly delineated by SPECT signal (Fig. 2A). Quantitative analysis of SPECT signal originating from the transplant revealed differences in ^111^In-exendin-3 accumulation depending on the number of initially transplanted islets, where the uptake was 5.9 kBq ± 2.4, 22.9 kBq ± 4.8, 30.1 kBq ± 10.1, 60.9 kBq ± 9.8 and 88.7 kBq ± 11.5, in muscles transplanted with 50, 100, 200, 400 and 800 islets, respectively (Fig. 2B). Immunohistochemical determination of graft volume was performed in all groups of mice (Fig. 2C). Plotting of SPECT data against the insulin staining volume revealed an excellent linear correlation between ^111^In-exendin uptake and transplant size (pearson *r* = 0.89).

## Discussion

The aim of this study was to evaluate the relation between ^111^In-exendin uptake in islet transplants compared to actual BCM in a muscle model of islet transplantation. Tracer uptake and beta-cell volume showed an excellent linear correlation. In addition, reliable visualization of transplants consisting of low numbers of islets indicates towards high sensitivity of this method. This high sensitivity in combination with the excellent correlation of BCM and tracer uptake demonstrate the potential of this method to quantify small differences in viable beta-cell volume.

Previously, imaging of liver islet transplants using ^64^Cu-labelled exendin has been demonstrated in NOD/SCID mice^19^. Twelve days follow-up of the grafts revealed significantly higher uptake of ^64^Cu-labeled exendin-4 in the liver of transplanted animals when compared to the control group. Here, we used the skeletal muscle transplantation as a highly controlled model for histological verification of the insulin positive area (which is challenging in the liver transplantation model given that islets are distributed throughout a large part of the organ), a prerequisite enabling us to, for the first time, reveal an excellent linear correlation between the actual numbers of beta-cells that were successfully engrafted, and the uptake of the tracer in the islet grafts.

Recently, it was reported that injection of ^123^I-IBZM enables the quantification of BCM in islet grafts, where tracer uptake could be linearly correlated with graft volume^23, 24^. However, a far lower correlation was observed between ^123^I-IBZM uptake and insulin positive graft volumes when compared to ^111^In-exendin, indicating that GLP-1R could allow more accurate assessments of islet graft survival. Moreover, our data indicate that ^111^In-exendin has a much higher sensitivity for detection of islet grafts when compared to ^123^I-IBZM as we could easily visualize islet grafts after transplantation of 50 islets while 1000 islets were needed for *in vivo* visualization by IBZM. In fact, more than 50% of the islets could be lost in the first days after transplantation^25^, indicating that ^111^In-exendin-SPECT was able to detect grafts containing far less than the 50 islets being initially transplanted. The superior detection sensitivity of ^111^In-exendin-SPECT could be explained by the higher abundance of the GLP-1R on the surface of the beta-cells when compared to the dopamine 2 receptor^21, 23^. Hence, ^111^In-exendin-SPECT has the potential to detect small grafts even after post transplantation beta-cell loss. This enables the possibility to evaluate and optimize treatment to preserve the remaining islets in patients that became insulin dependent after islet transplantation, helping to preserve their positive effect on glucose homeostasis.

The skeletal muscle was selected as a transplantation site to evaluate whether ^111^In-exendin-3 can be used to monitor beta-cell volume after transplantation. So far, this site has been used for islet transplantation in an experimental setting in small animal models and in a few patients^20^. In the clinical setting, intra-portal islet transplantation still predominates^26^. The ability to detect islet transplants in the liver with exendin-3 has previously been demonstrated in a proof of concept study in mice^19^. Further studies are warranted to assess whether our finding that the ^111^exendin-3 uptake corresponds to the beta-cell volume after transplantation is also valid after intrahepatic islet transplantation.

In conclusion, we have demonstrated for the first time that ^111^In-exendin-3 can be used to determine beta-cell volume after intramuscular islet transplantation. ^111^In-exendin-3 uptake linearly correlates with the amount of living beta-cells, and allows the detection of islet grafts consisting of less than 50 islets by SPECT imaging. Our data indicates that this approach holds great potential for accurate and sensitive quantification of viable beta-cells in a transplantation setting. Clinical studies evaluating the potential of this promising radiotracer for imaging of islets grafts in humans are under preparation.

## Research Design and Methods

### Animals

Female C3H/HeNCrl mice (22–30 g) were purchased from Charles River (Calco, Itlay). All experiments were conducted in accordance with Radboud University guidelines on humane care and use of laboratory animals and were approved by the Animal Ethical Committee of the Radboud University, Nijmegen, The Netherlands.

### Pancreatic islet isolation and transplantation

Pancreatic islets were isolated from 6–8 weeks old mice by a collagenase digestion method. Briefly, mice were euthanized and 2 ml of cold RPMI 1640 (Invitrogen, Carmarillo, CA, USA) containing collagenase type V (1 mg/ml; Sigma Aldrich, St Louis, MO, USA) were infused into the pancreatic duct *in situ*. Perfused pancreata were collected in serum-free RPMI medium and kept on ice until enzymatic digestion at 37 °C for 12 min. Islets were purified on a discontinuous Ficoll gradient of following densities: 1.118, 1.096 and 1.037 g/ml (Cellgro by Mediatech Inc., Manassas, VA, USA) and islets were collected between the second and the third fraction. Islets were cultured overnight in a humidified 5% CO_2_ atmosphere at 37 °C in RPMI 1640 medium supplemented with L-glutamine (Sigma Aldrich, St. Louis, MO, USA), penicillin-streptomycin (10 mg/ml; Sigma Aldrich) and 10% (v/v) fetal calf serum (HyClone, Celbio, Logan, UT, USA). Islets were counted and hand-picked under bright field microscope and 50, 100, 200, 400 or 800 islets were transplanted in the calf muscle (n = 5 per group), parallel to the fibula, using needles with a 0.8 mm diameter. The exact number of transplanted islets was determined by subtracting the remaining islets in the tube from the initially counted number.

### Radiolabeling of exendin-3

[Lys ^40^(DTPA)]-exendin-3 was purchased from Peptide Specialty Laboratories (Heidelberg, Germany). Tracer labeling with In-111 was performed as previously described^17^. Radiochemical purity was determined by ITLC and radiolabeled exendin-3 was purified by solid-phase extraction using a HLB-column as previously reported^17^.

### SPECT acquisition

Mice with 50, 100, 200, 400 and 800 islets were scanned after 4 weeks. All mice (n = 4–5) were injected with approximately 15 MBq of ^111^In-exendin-3 (peptide dose 0.1 µg in 200 µl PBS, 0.5% BSA) in the tail vein. SPECT scans were acquired 1 h post-injection on a U-SPECT-II/CT dedicated small-animal scanner (MILabs, Utrecht, Netherlands) for 50 min with a high sensitivity mouse collimator (1.0 mm pinholes). Computed tomography (CT) was performed subsequently for anatomical reference. Standards of 74 kBq, 55 kBq, 37 kBq and 18 kBq in 50 µl volume each, were scanned under the same parameters as reference for quantification. Images were reconstructed with voxel size of 0.4 mm, 3 iterations and 16 subsets, using U-SPECT-II reconstruction software (MILabs, Utrecht, The Netherlands). The VOI was drawn over the islet transplant region, total voxel intensity registered in the islet graft was corrected by the mean of 3 measurements of contra-lateral control muscle, to subtract the background signal originating from the muscle tissue. The absolute activity (in kBq) was calculated by multiplying the corrected voxel intensity value with the calibration factor determined by quantitative analysis of standards with known radioactivity and data were normalized by the injected dose.

### Morphometric analysis of the transplant

Immediately after SPECT acquisitions, mice were euthanized, muscles were fixed in 4% paraformaldehyde and embedded in paraffin, then sectioned into 4 µm slices for autoradiography analysis or for determination of insulin volume by immunohistochemistry. For autoradiography analysis, muscle sections were exposed to an imaging plate (Fuji Film BAS-SE 2025, Raytest, Straubenhardt, Germany) for 7 days and images were visualized with a radioluminography laser imager (Fuji Film BAS 1800 II system, Raytest, Straubenhardt, Germany) and were finally stained with hematoxylin-eosin (HE) to confirm the presence of islets. For determination of insulin volume, insulin staining was performed in muscle sections. Antigen retrieval was done using 10 mM sodium citrate buffer, pH 6.0, for 10 min (Thermo Scientific PT module, Lab Vision, USA). Blocking of endogenous peroxidase activity was performed by incubation with 0.6% H_2_O_2_ in 40% methanol/60% PBS for 30 min at RT in the dark. An additional blocking step was done with 5% swine serum in PBS for 30 min at RT. Primary anti-insulin antibody (cat. sc 9168, Santa Cruz Biotechnology, Inc., Santa Cruz, CA, USA) was applied at a dilution of 1:50 (in PBS containing 1% BSA w/v). Subsequently, sections were washed with PBS and incubated with secondary horseradish peroxidase-conjugated swine-anti-rabbit IgG (1:50) (cat. P0217, Dakopatts, Copenhagen, Denmark) in PBS containing 1% BSA w/v for 30 min at RT. The staining was visualized with diaminobenzidine (PowerVision^TM^ DAB substrate system, Immunologic, Duiven, The Netherlands) and nuclei were counterstained with haematoxylin. To determine the volume of the transplant, sections were scanned with Pannoramic250 Flash II scanner (3D Histech, Budapest, Hungary), beta-cell surface was manually drawn around insulin positive region using Photoshop CS6. Finally, the volume was determined by multiplying the insulin positive surface per section by the inter-section distance, which is 40 µm.

### Statistical analysis

Statistical analyses were performed using with Graphpad Prism 5 (San Diego CA, USA). The results were presented as mean ± SEM. Correlations were assessed using a two-tailed Pearson’s correlation coefficient. The level of significance was set at *P* < 0.05.
